# Emerging pesticides responsible for suicide in rural Sri Lanka following the 2008–2014 pesticide bans

**DOI:** 10.1186/s12889-020-08871-7

**Published:** 2020-05-25

**Authors:** Manjula Weerasinghe, Melissa Pearson, Flemming Konradsen, Suneth Agampodi, J. A. Sumith, Shaluka Jayamanne, S. M. H. M. K. Senanayake, Sandamali Rajapaksha, Michael Eddleston

**Affiliations:** 1 Department of Community Medicine, Faculty of Medicine & Allied Sciences, Rajarata University of Sri Lanka, Anuradhapura, Sri Lanka; 2 South Asian Clinical Toxicology Research Collaboration, Faculty of Medicine, University of Peradeniya, Peradeniya, Sri Lanka; 3 Centre for Pesticide Suicide Prevention, and Pharmacology, Toxicology and Therapeutics, Centre for Cardiovascular Science, University of Edinburgh, Edinburgh, UK; 4 Department of Public Health, Faculty of Health and Medical Sciences, University of Copenhagen, Copenhagen, Denmark; 5 Office of the Registrar of Pesticides, Getambe, Peradeniya, Sri Lanka; 6 Department of Medicine, Faculty of Medicine, University of Kelaniya, Ragama, Sri Lanka; 7 Teaching Hospital Anuradhapura, Anuradhapura, Sri Lanka

**Keywords:** Pesticide, Pesticide regulation, Self-poisoning, Suicide, Sri Lanka

## Abstract

**Background:**

Sri Lanka has reduced its overall suicide rate by 70% over the last two decades through means restriction, through a series of government regulations and bans removing highly hazardous pesticides from agriculture. We aimed to identify the key pesticide(s) now responsible for suicides in rural Sri Lanka to provide data for further pesticide regulation.

**Methods:**

We performed a secondary analysis of data collected prospectively during a cluster randomized controlled trial in the Anuradhapura district of Sri Lanka from 2011 to 16. The identity of pesticides responsible for suicides were sought from medical or judicial medical notes, coroners’ records, and the person’s family. Trend analysis was done using a regression analysis with curve estimation to identify relative importance of key pesticides.

**Results:**

We identified 337 suicidal deaths. Among them, the majority 193 (57.3%) were due to ingestion of pesticides while 82 (24.3%) were due to hanging. A specific pesticide was identified in 105 (54.4%) of the pesticide suicides. Ingestion of carbosulfan or profenofos was responsible for 59 (56.2%) of the suicides with a known pesticide and 17.5% of all suicides. The increasing trend of suicides due to carbosulfan and profenofos over time was statistically significant (R square 0.846, F 16.541, p 0.027).

**Conclusion:**

Ingestion of pesticides remains the most important means of suicides in rural Sri Lanka. The pesticides that were once responsible for most pesticide suicides have now been replaced by carbosulfan and profenofos. Their regulation and replacement in agriculture with less hazardous pesticides will further reduce the incidence of both pesticide and overall suicides in rural Sri Lanka.

## Background

Ingestion of pesticides is one of the three most important global means of suicide [1], accounting for up to 20% of global suicides [2]. Widespread agricultural use of highly hazardous pesticides [3] (HHP) in small-scale farming in low and middle income countries (LMIC), poor home storage [4], and unrestricted over-the-counter sales [5] ensure that such pesticides are easily accessible for acts of self-poisoning. Our recent systematic review indicates that a worldwide ban on the use of HHP in agriculture is likely to be the most effective means of reducing both pesticide-specific and overall suicide rates in countries where pesticides are important means of suicide [6].

Pesticide self-poisoning was the most common means of suicide in Sri Lanka during 1976 to 2011 [7]. However, Sri Lanka has used means restriction and pesticide regulation to sequentially ban agricultural pesticides that have become popular for self-harm [8] producing one of the greatest falls in suicide ever seen [9]. Knipe and colleagues recently estimated that Sri Lanka has reduced its overall suicide rate by 70% (saving 93,000 lives) since 1995 predominantly through regulation of pesticides [10]. This was done despite being unable to comprehensively address [6] the complex psychosocial and cultural factors that may underlie suicide [1]. As a result, the WHO recently published, with the FAO, a guide for pesticide regulators on suicide prevention [11] and recently proposed that regulation of problematic highly hazardous pesticides is a highly cost-effective approach to suicide prevention [12].

However, pesticide self-poisoning is still the second most common method of suicide in Sri Lanka, causing 800 to 1000 deaths for each of the last 3 years according to official statistics [13]. Most recent data suggested that the incidence of pesticide suicide in rural Sri Lanka remains around 20/100,000 population [14] – far higher than the global incidence of suicide from all means [1] Many of these deaths occur after ingestion of pesticides classified by the WHO as being of only moderately acute toxicity [15], due to the large volumes ingested and the solvents in these agricultural formulations [16, 17].

A prospective cohort study conducted in secondary hospitals [18] (2002–2008) reported that > 78% of pesticide suicides were due to five pesticides (the organophosphorus (OP) insecticides dimethoate, fenthion, and chlorpyrifos, and the herbicides paraquat and propanil). Although the case fatality was higher for dimethoate, fenthion, and paraquat, which were responsible for most deaths, the common use of the other less hazardous pesticides (chlorpyrifos and propanil) meant that they were also responsible for many suicides [18]. In 2008, Sri Lankan regulatory authorities made a decision to remove dimethoate, fenthion, and paraquat from agriculture over 3 years to reduce suicides [19]. This was followed by withdrawal of chlorpyrifos and propanil in 2014 [20]; of note, these latter withdrawals were not focused on suicide prevention but on other potential health issues [21].

Withdrawal of one agricultural pesticide usually results in its replacement by another product targeting the same pest or weed (or of a non-chemical approach to pest control); therefore, continued monitoring is required to identify new pesticides rising in popularity for agriculture and hence suicide in a timely manner [10, 22]. Therefore, we have continued to monitor the pesticides responsible for fatal self-harm since 2011. In this work, we aimed to identify the key pesticide(s) currently responsible for pesticide suicides in rural Sri Lanka to inform decisions about the need for future pesticide regulations.

## Methods

### Study design

**Fig. 1:**
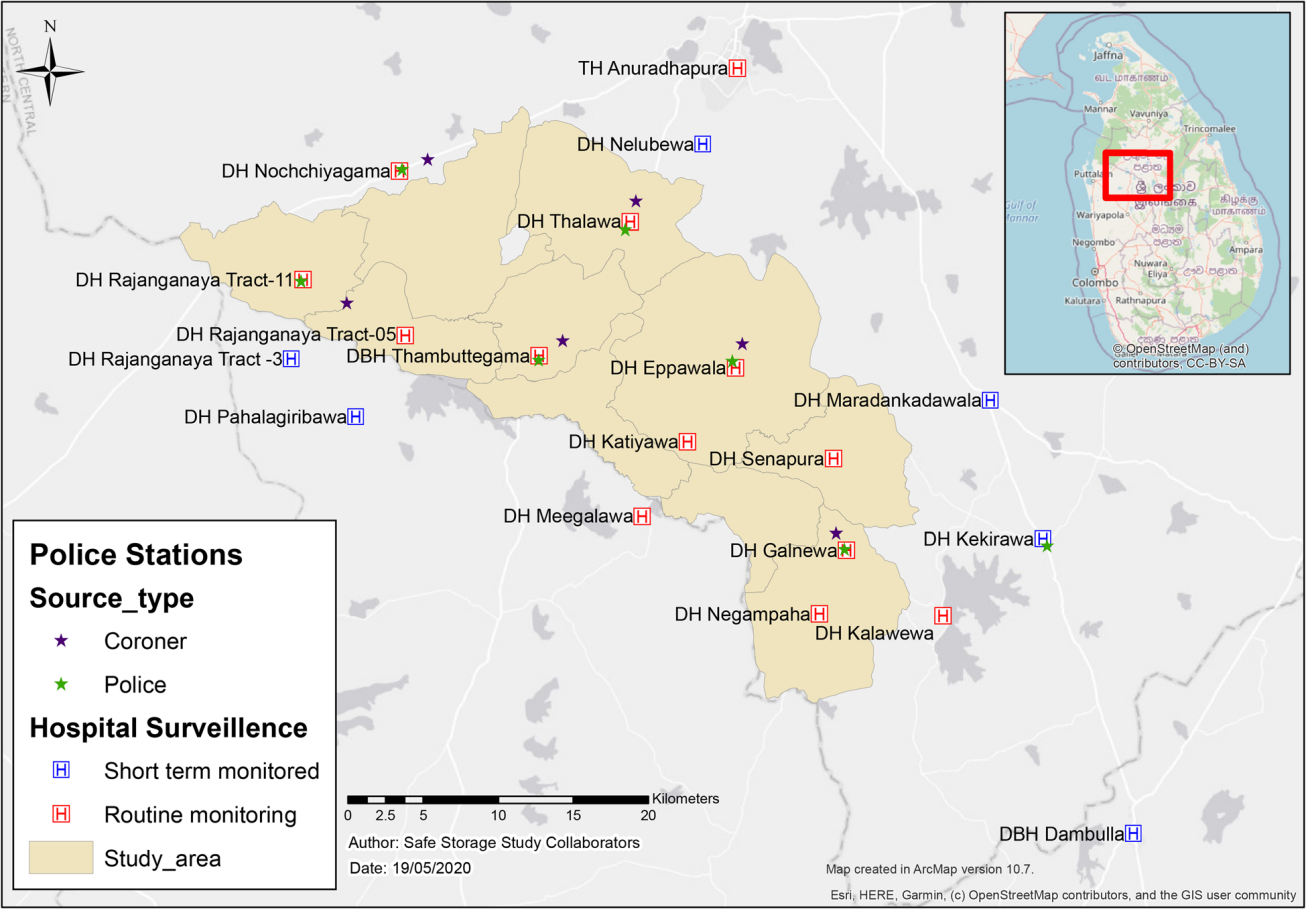
Study area in south western Anuradhapura district, Sri Lanka, showing the hospitals, police stations and coroner courts from which data were collected. Created for this publication in ArcMap version 10.7 under personal license by the authors

This study reports a secondary analysis of prospective suicide data collected among more than 250,000 population residing in six divisional secretariats (Thambuttegama, Thalawa, Nochchiyagama, Rajanganaya, Galnewa and Ipalogama) of south western Anuradhapura District, North Central Province of Sri Lanka (Fig. 1) as part of a cluster-randomized controlled trial [14]. The population under study varied across the study period; therefore, no estimate of incidence has been calculated from the data.

### Primary dataset and data collection procedure

Data used in this study were collected during post-intervention follow-up surveillance for a community-based cRCT to evaluate the effectiveness of household pesticide storage devices for reducing pesticide self-poisoning [14, 23]. Data on suicides due to pesticide poisoning was collected between July 2011 and May 2016. Data were collected from six different local sources where deaths are registered (Table 1) and cross-checked, to reduce the risk that suicides would be missed or wrongly reported during data collection period.

**Table 1:** Death registration sources and their network in the study area

Data source	Network
Hospitals (*n* = 13)	DBH Thambuttegama, DH Thalawa, DH Nochchiyagama, DH Eppawala, DH Rajanganaya tract-11, DH Rajanganaya tract-05, DH Katiyawa, DH Senapura, DH Negampaha, DH Galnewa, DH Kalawewa, DH Meegalewa, Teaching Hospital Anuradhapura
Judicial Medical Officer (*n* = 1)	Teaching Hospital Anuradhapura
Police (*n* = 7)	Thambuttegama, Thalawa, Eppawala, Nochchiyagama, Rajanganaya, Galnewa, Kekirawa
Community coroners (*n*- = 10)	Uthuru wilachchiya coralaya, Dakunu wilachchiya coralaya, Kalankuttiya coralaya, Nagampaha coralaya, Pahalagama coralaya, Rajanganaya coralaya, Eppawala coralaya, Jayasiripura coralaya, Lidawewa coralaya, Eliyadiwulwewa coralaya
Divisional Secretariats (*n* = 6)	Thambuttegama, Thalawa, Eppawala, Nochchiyagama, Rajanganaya, Galnewa
Court (*n* = 1)	Magistrate’s court - Thambuttegama

Abbreviations: *DBH* district base hospital, *DH* district hospital

Information on deaths occurring in primary and secondary hospitals was extracted daily from the episodic medical notes (termed bed head tickets; BHT) and/or ward death registers, whereas in tertiary hospitals information was extracted from Judicial Medical Officer (JMO) records at the end of the data collection period. Deaths that occurred before hospital presentation were identified by regular review of the records of community coroners and police. At the end of the data collection period, suspected suicide deaths and deaths with open decisions were sought by examination of records belonging to local magistrates. Finally, copies of death certificates at the divisional secretariats were manually searched to minimize the number of suicides missed in the prospective data collection.

Data were extracted from death records using a structured data collection form by trained research assistants. If necessary, additional visits were made by the research team to the dead person’s family or relatives to collect missing information.

Underlying causes of death are coded in the data according to the International Classification of Diseases, 10th Revision (ICD-10) [24]. Suicide by pesticide poisoning was defined as those cases featuring the ICD-10 code X68 and intentional self-harm by hanging, strangulation or suffocation is X70. Among suicides due to pesticide poisoning, ICD-10 T codes were used to identify the specific category of agents including insecticides (T60.0-T60.2), herbicides and fungicides (T60.3), rodenticides and other pesticides (T60.4, T60.8) and unspecified pesticides (T60.9).

### Definition of a pesticide suicide

A pesticide suicide was defined as an act of deliberately killing oneself by ingesting one or more agricultural pesticides (insecticides, herbicides, fungicides, and rodenticides) with the case being confirmed and registered in one or more of the death registration sources during the study period.

### Identification of ingested pesticide

For hospitalized patients, the ingested pesticide was normally reported in the BHT or JMO’s report, based on the history from patients or family, seeing the pesticide product label or bottle, and/or clinical symptoms and post-mortem examination. For non-hospitalized cases, this information was obtained from police and/or coroners’ records. If the identity of the ingested pesticide was not available in any official sources, the research team re-contacted the dead person’s family soon after the death to identify the pesticide container. If there was inconsistency of the ingested pesticide(s) between the two or more sources, the medical examination report was given priority.

### Data linking

After collection of death records from sources, data were linked manually using a simple algorithm which used death persons’ demographic information (name, age, gender, address) and incident related information (date/time of incident, date/time of death, type/active ingredient of the ingested pesticide) to identify multiple records of the same event and prevent double counting. After cases were identified, they were allocated a unique case identifier.

### Data analysis

We used GraphPad Prism, Quickcalcs and SPSS 25 for analyses. Descriptive statistics were used to describe the types of pesticides and types of active ingredients. Trend analysis was done using a regression analysis with curve estimation.

## Results

### Pesticide suicides

**Fig. 2:**
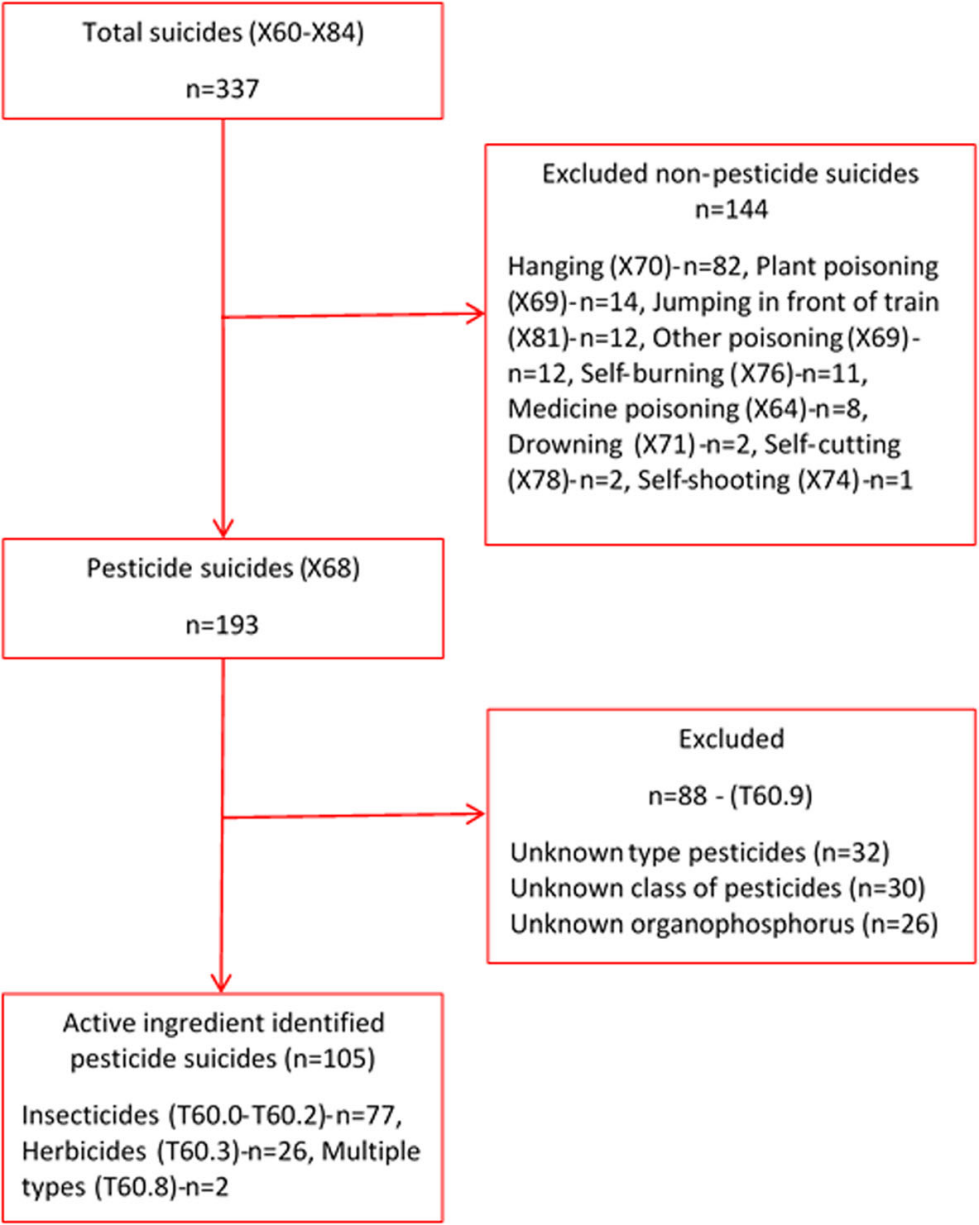
Flow chart of eligible pesticide suicide cases identified from rural Anuradhapura, Sri Lanka from 2011 to 2016 period

The data identified a total of 337 suicidal deaths. Of these cases, 193 (57.3%) and 82 (24.3%) were reported as being due to ingestion of pesticides and hanging, respectively (Fig. 2). The type of pesticide was unknown in 32/193 (16.6%) cases, the class of pesticide was unknown in 30/193 (15.5%) cases, and the particular OP insecticide was unknown in 26/193 (13.5%) cases. Where the pesticide type was known, 125/161 (77.6%) were due to insecticides, 31/161 (19.3%) due to herbicides, and 5/161 (3.1%) due to other type of pesticides. OP insecticides were responsible for 50/131 (38.2%) pesticide suicides with identified pesticides over the study period. The following analysis includes only the 105 cases for which the specific pesticide was positively identified.

### Common pesticides used for suicide in 2011–2016

**Fig. 3:**
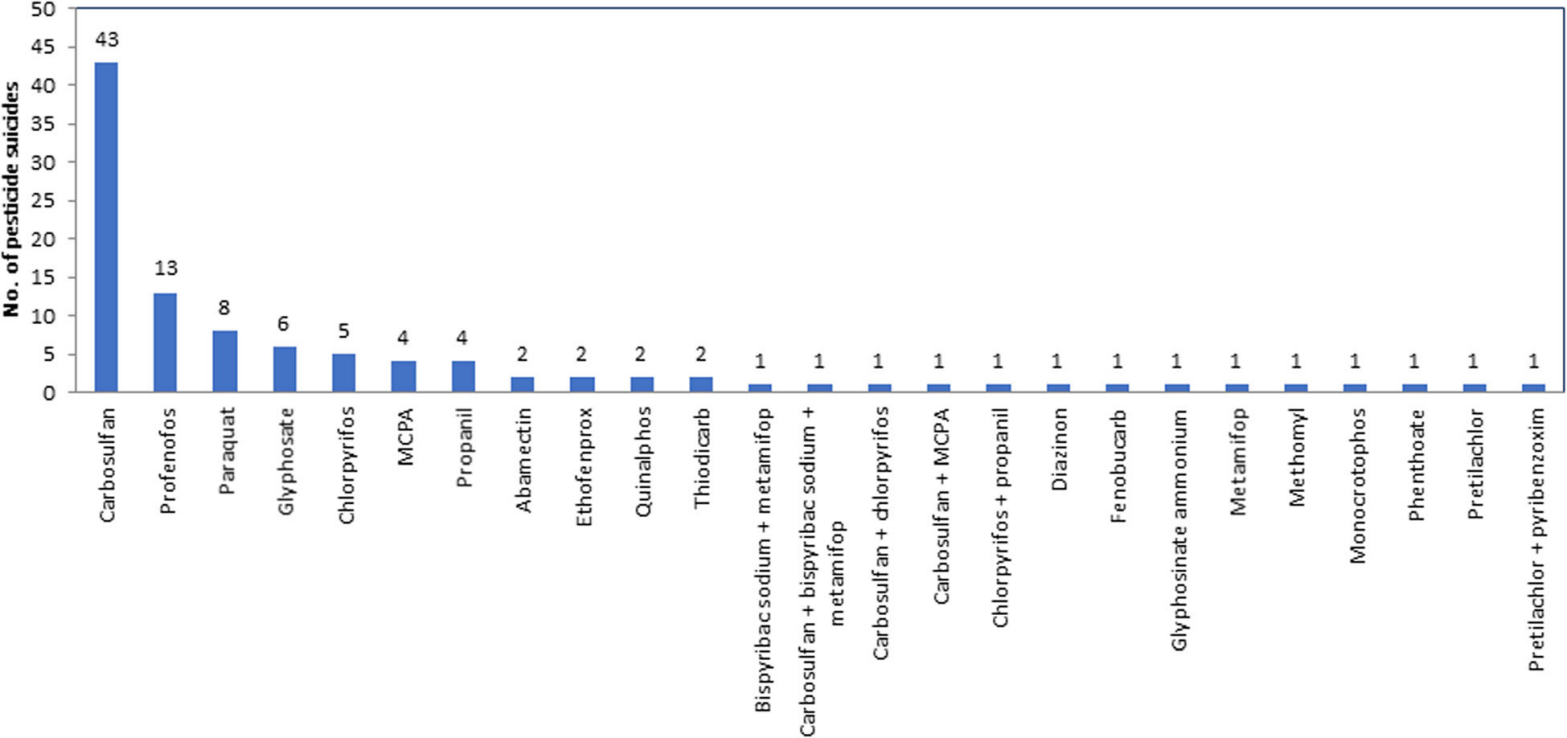
Pesticides identified as being responsible for suicides during 2011–2016 period from rural Anuradhapura, Sri Lanka

The majority of deaths resulted from ingestion of the carbamate insecticide carbosulfan (43, 41.0%) or the OP insecticide profenofos (13, 12.4%) (Fig. 3). Carbosulfan was involved in a further three mixed pesticide self-poisoning cases (giving a total of 46 cases, 43.8%). Ingestion of carbosulfan or profenofos was directly implicated in 56.2% [59/105] pesticide suicides (and at least 17.5% [59/337] of overall suicides). Over the same period three pesticides banned in 2008 and 2014 (paraquat, chlorpyrifos and propanil) were involved in only 17% (18/105) pesticide suicides (and at least 5.3% of all means of suicides). Dimethoate and fenthion were not reported as being involved in any suicides during 2011 to 2016. There were very few cases of suicides with pesticides banned before 2008 (Fig. 3).

### Relative importance of pesticides over time

**Fig. 4:**
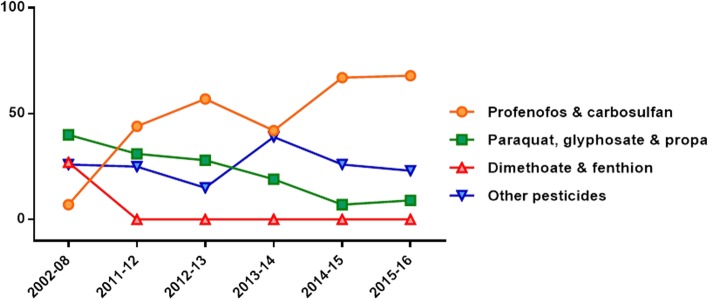
Key pesticides responsible for suicides in 2002–2008 vs 2011–2016 Anuradhapura, Sri Lanka

The proportion of suicides due to carbosulfan or profenofos increased steadily from 2011 to 2016 from 43.8 to 68.1% (Fig. 4). During the same period, the proportion of deaths due to paraquat, chlorpyrifos and propanil fell from 25.0 to 13.6%. The increasing trend of suicides due to carbosulfan and profenofos was statistically significant (R square 0.846, F 16.541, p 0.027).

## Discussion

Nationally, hanging is now the most common method of suicide in Sri Lanka [7, 13]. However, our data reveal that in rural agricultural parts of Sri Lanka where pesticides are widely used and available, they remain more than twice as important as hanging for suicide. Notably, this data set identifies the insecticides carbosulfan and profenofos to be the key pesticides involved in suicides, likely responsible for more than half of pesticide suicides and almost 1 in 5 of all suicides. Many of the unknown fatal OP or carbamate insecticide poisoning cases are likely to be due to these commonly used insecticides as well.

The pattern of pesticides responsible for suicides changed markedly between 2002 and 08 [18] and 2011–16 (Fig. 4). In 2002–08 and in 2011–16, the proportion of suicides due to herbicides deceased from 45 to 25% whereas suicides due to insecticides increased from 54 to 75% respectively. In 2002–08, carbosulfan and profenofos were responsible for 7% of pesticide suicides in a large prospective secondary hospital cohort in the North Central Province of Sri Lanka [18]. However, ingestion of carbosulfan or profenofos was responsible for 59 (56.2%) of the suicides for the period of 2011–16.

A recent hospital-based study from Peradeniya, in central Sri Lanka, identified profenofos as a key cause of death but also multiple long intensive care admissions due to the OP insecticide quinalphos [25]. However, although cases of quinalphos self-poisoning have been noted in North Central Province [18], few deaths were reported in this study - perhaps because quinalphos is not a commonly used insecticide among local farmers.

It is important to know why carbosulfan and profenofos are such commonly used in suicides in recent years. A study conducted by Rathish and colleagues reported that carbosulfan and profenofos were the two highest selling pesticides in local rural shops after 2015 [26]. One explanation is that the withdrawal from agriculture of dimethoate and fenthion over 3 years (2008–2011) followed by chlorpyrifos in 2014 has resulted in a rapid increase in use of carbosulfan and profenofos in agriculture and then suicide. In addition, the importance of carbosulfan and profenofos for suicide is probably because of their relatively higher case fatality [27, 28] compared to other remaining pesticides [18].

Of note, although the suicide rate in Sri Lanka is continuing a downward trend with these recent bans [10], the reduction would be faster still if chlorpyrifos had not been banned and this relatively less toxic OP compound had remained the most common insecticide used in agriculture, and therefore in self-harm. The herbicide that appears to have replaced glyphosate is diuron (Weerasinghe, unpublished observations) which is of low acute toxicity (WHO toxicity class III slightly hazardous) [15] and was not involved in the suicides reported here.

We noted several suicides due to paraquat, chlorpyrifos, glyphosate or propanil during the study period, with a few occurring after their bans in 2011 and 2014. This is likely due to patients ingesting pesticides stored in the farming households or bought after the ban, as stocks ran down in local shops. However, previous studies suggest that bans will result in pesticides being no longer used for suicide within a few years [29]. Our data also supported this – no deaths involving dimethoate or fenthion occurred in this study period (Fig. 4).

Previous studies in Sri Lanka [19, 30], Bangladesh [31] and South Korea [32] provide no evidence that pesticide bans result in reduced agricultural yield and/or increased input costs to the farmer. However, pesticide regulations need to be carefully planned to reduce potential adverse effects on agricultural output and costs, with support of integrated pest management and replacement of highly hazardous pesticides with less hazardous compounds. According to Sri Lanka’s manual of pesticide management recommendation, profenofos is the only recommended insecticide for gall fly *(Neolasioptera falcata)* in Cucurbits [33]. Therefore, studies are required to identify substitutes and alternative strategies for pest control before regulation of such insecticide.

A common concern about limiting access to one lethal method used for suicide (means restriction) is that individuals will simply switch to other methods of suicide [34]. For example, regulation of selected pesticides might result in method substitution, possibly to methods of suicide with higher case fatality (such as hanging). However, the data from Sri Lanka and other countries [6] indicates that individuals simply switch to other, less toxic pesticides, resulting in fewer deaths from pesticides poisoning and any forms of suicide. In addition, withdrawing two pesticides from agricultural practice does not impact for pesticide availability for self-harm as many other pesticides are available in the market. The evidence for switching to more lethal method is not strong in Asia [35].

Suicide prevention efforts will need to be multifaceted [36]. In addition to regulation efforts, community-based prevention through public health interventions is especially important to reduce acts of self-poisoning. Over the last decade, a considerable attention has been given to public health approaches in Sri Lanka, such as improved household storage and vendor-based sales restrictions. Unfortunately, our recent cluster randomized controlled trial showed no reduction in pesticide self-poisoning as a result of improved pesticide storage [14]. Vendor-based sales restrictions are now being piloted, but no evidence currently exists on the effectiveness of this approach [36]. Multiple structural and social factors are associated with suicide in South Asia (for example: debt, poverty, cash cropping, intimate partner violence, and alcohol) [37]. However, these risk factors are more diffuse and would require a large policy consensus for action. The best evidence currently indicates that national pesticide regulation is likely to be more effective than community-level approaches [9].

Identifying the pesticides most commonly used in fatal self-poisoning and taking regulatory actions to them phase out is a key WHO and FAO recommendation to reduce pesticide suicides [11]. One of the core reasons for the effective reduction pesticide suicides in Sri Lanka is continuous monitoring of key pesticides used in suicides followed by timely policy decisions – for example 2000–11 withdrawal of the three pesticides (dimethoate, fenthion, and paraquat) that were responsible for the majority of pesticide deaths in the 2000s [10]. This analysis shows that the recent bans resulted in a switch to carbosulfan and profenofos. Therefore, ongoing monitoring of pesticide suicide data is essential to implement regulatory decisions in a timely manner. These lessons learned from Sri Lanka are relevant to other countries where pesticide suicides are more common.

### Limitations

The main limitation is the lack of data on the pesticide ingested in 46% of cases due to the lack of forensic toxicology results with which to confirm exposure. However, we hypothesize that many of these deaths will have been due to carbosulfan poisoning since this insecticide has a relatively fast onset of poisoning [28], causing death before presentation to hospital where data on the pesticide ingested was best collected. However, we have generally found the history to be very accurate for identifying the pesticide where blood analyses have been done retrospectively [27, 28].

Our data are from one part of the island; there is no parallel sentinel monitoring of pesticide suicide in other parts of Sri Lanka. However, the area is typical for other agricultural regions of Sri Lanka and it is likely that the findings are generalizable to rural Sri Lanka generally.

## Conclusion

Ingestion of pesticides remains the most common means of suicide in rural Sri Lanka. Withdrawal of key agricultural pesticides (dimethoate, fenthion, paraquat, chlorpyrifos, and propanil) due to a series of bans in 2008–14 resulted in a pesticide suicide switch to carbosulfan and profenofos. Although less acutely toxic than pesticides previously associated with suicides, these insecticides remain highly hazardous after ingestion. These insecticides had a higher case fatality after overdose than the chlorpyrifos insecticide they replaced, becoming responsible for many deaths. The herbicides that took over from paraquat, then glyphosate, appeared to be safe for self-harm as no cases were recorded by 2015–16. Regulating carbosulfan and profenofos will result in effective prevention of both pesticide-specific and overall suicides in the North Central Province and likely across rural Sri Lanka.

## Data Availability

The anonymous dataset used for the current study is available upon request from the corresponding author.

## Abbreviations

BHT: Bead Head Ticket
cRCT: Cluster Randomized Controlled Trail
HHP: Highly Hazardous Pesticides
ICD: International Classification of Diseases
JMO: Judicial Medical Officer
LMIC: Low- and Middle-Income Countries
OP: Organophosphates
WHO: World Health Organization
